# TDP-43 and ER Stress in Neurodegeneration: Friends or Foes?

**DOI:** 10.3389/fnmol.2021.772226

**Published:** 2021-10-25

**Authors:** Lorena de Mena, Joshua Lopez-Scarim, Diego E. Rincon-Limas

**Affiliations:** ^1^Department of Neurology, McKnight Brain Institute, and Norman Fixel Institute for Neurological Diseases, University of Florida, Gainesville, FL, United States; ^2^Department of Neuroscience, Center for Translational Research in Neurodegenerative Disease, University of Florida, Gainesville, FL, United States; ^3^Genetics Institute, University of Florida, Gainesville, FL, United States

**Keywords:** TDP-43, ER stress, UPR, neurodegeneration, proteinopathies, pERK, XBP1

## Abstract

Nuclear depletion, abnormal modification, and cytoplasmic aggregation of TAR DNA-binding protein 43 (TDP-43) are linked to a group of fatal neurodegenerative diseases called TDP-43 proteinopathies, which include amyotrophic lateral sclerosis (ALS) and frontotemporal lobar degeneration (FTLD). Although our understanding of the physiological function of TDP-43 is rapidly advancing, the molecular mechanisms associated with its pathogenesis remain poorly understood. Accumulating evidence suggests that endoplasmic reticulum (ER) stress and the unfolded protein response (UPR) are important players in TDP-43 pathology. However, while neurons derived from autopsied ALS and FTLD patients revealed TDP-43 deposits in the ER and displayed UPR activation, data originated from *in vitro* and *in vivo* TDP-43 models produced contradictory results. In this review, we will explore the complex interplay between TDP-43 pathology, ER stress, and the UPR by breaking down the evidence available in the literature and addressing the reasons behind these discrepancies. We also highlight underexplored areas and key unanswered questions in the field. A better synchronization and integration of methodologies, models, and mechanistic pathways will be crucial to discover the true nature of the TDP-43 and ER stress relationship and, ultimately, to uncover the full therapeutic potential of the UPR.

## Introduction

The abnormal distribution, modification, and aggregation of TAR DNA-binding protein 43 (TDP-43) are the pathological hallmarks of a group of neurodegenerative diseases that are collectively known as TDP-43 proteinopathies (Tziortzouda et al., 2021). In these disorders, the nuclear depletion and cytoplasmic accumulation of TDP-43 leads to gain- and loss-of-function abnormalities within neurons by altering a wide range of biological processes including RNA biogenesis, autophagy, the ubiquitin proteasome system (UPS), and axonal transport among others, that ultimately determine cell survival (Prasad et al., 2019). Despite major advances in understanding the physiological role of TDP-43, we still have very limited knowledge of the precise molecular and cellular mechanisms of pathogenesis induced by neurotoxic TDP-43 species. A key challenge, therefore, is to identify critical targets and pathways mediating neuronal degeneration. In this regard, recent evidence suggests that endoplasmic reticulum (ER) stress may play a pivotal role in the development or pathogenesis of TDP-43 proteinopathies (Walker and Atkin, 2011). Accordingly, a major proteostatic pathway known as the Unfolded Protein Response (UPR) seems to respond to TDP-43 aggregation-induced ER stress by guiding proadaptive and/or proapoptotic cell fate decisions. However, there are several discrepancies and controversies in this regard that should be taken into consideration. Thus, in this review, we aim to shed light on the complex relationship existing between TDP-43, ER stress, and the UPR in the context of neurodegeneration.

## TDP-43 Functions

**Figure 1:**
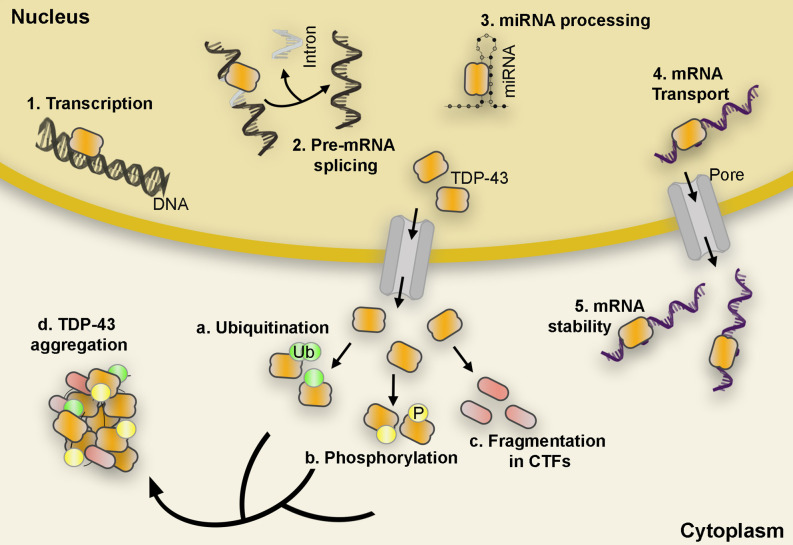
TDP-43 functional roles and post-translational modifications. TDP-43 carries out a variety of functions such as initiation of transcription, pre-mRNA splicing, miRNA processing, mRNA transport, and mRNA stability **(1–5)**. However, in pathological conditions, TDP-43 is depleted from the nucleus and accumulates in the cytoplasm in hyperphosphorylated, ubiquitinated, and cleaved (CTFs) forms found in the abnormal protein aggregates characteristic of ALS and FTLD proteinopathies **(a–d)**. Abbreviations: mRNA, messenger RNA; miRNA, microRNA; CTF, C-terminal fragments; ALS, amyotrophic lateral sclerosis; FTLD, Frontotemporal lobar degeneration; Ub, Ubiquitin; P, Phosphate.

TDP-43 is a ubiquitously expressed and multifaceted 414-amino acid RNA/DNA binding protein that is predominantly localized in the nucleus where it carries out a vast range of cellular functions involving mRNA splicing, stability, maturation, and transport, as well as transcriptional repression (Ayala et al., 2008). However, in TDP-43 proteinopathies, these processes are compromised or turned aberrant as nuclear TDP-43 is depleted, mislocalized, and modified to generate insoluble cytoplasmic inclusions marked by hyperphosphorylation, ubiquitination, and fragmentation (Figure 1). The formation of these inclusions coincides with an increase in neuronal dysfunction that underlies neurodegeneration in several diseases including amyotrophic lateral sclerosis (ALS) and the most common subtype of frontotemporal lobar degeneration (FTLD-TDP; Arai et al., 2006; Neumann et al., 2006; Hasegawa et al., 2008; Igaz et al., 2008). Loss of TDP-43 nuclear function is reported to disrupt cellular homeostasis; impede axonal transport by mRNA microtubule-dependent impairment (Alami et al., 2014); induce abnormal mitochondrial distribution, localization, and density (Wang et al., 2016); produce a surge in cryptic exon splicing (Ling et al., 2015); and alter ER function (*to be discussed in a later section*). Accumulation of excess or abnormally localized TDP-43 contributes to an increase in cytoplasmic aggregation that leads to the sequestration of nuclear TDP-43, other off-target proteins, and mRNAs from the environment resulting in a toxic gain of function (Barmada et al., 2010; Dammer et al., 2012; Lee et al., 2012; Zhang et al., 2013; Cascella et al., 2016; Russo et al., 2017). In addition, many genetic mutations in the gene encoding the TDP-43 protein, TARDBP, associate with familiar forms of ALS and FTLD-TDP although their incidence only accounts for ~4% of the familial ALS (fALS) diagnosed cases (Jo et al., 2020). Both nuclear depletion and overexpression of TDP-43, wild type (WT) and mutant, often lead to cell toxicity *in vitro* and *in vivo* models (Kabashi et al., 2009; Diaper et al., 2013; Cascella et al., 2016). This underscores the involvement of both loss- and gain-of-function mechanisms in disease pathogenesis.

### TDP-43 Modifications

Phosphorylation, ubiquitination, and aberrant cleavage are the most significant post-translational modifications found in TDP-43 protein inclusions and are believed to associate with the pathogenic alterations observed in ALS and FTLD patients (Neumann et al., 2006; Hasegawa et al., 2008; Igaz et al., 2008; Kametani et al., 2016; Buratti, 2018).

Although the definitive pathological role of phosphorylation still remains unknown, substantial evidence suggests that TDP-43 hyperphosphorylation might promote neurotoxicity, increase TDP-43 oligomerization, mislocalization, and seeding (Hasegawa et al., 2008; Liachko et al., 2010, 2014; Nonaka et al., 2016; Goh et al., 2018; Taylor et al., 2018). For instance, inhibition of Casein Kinase 1 (CK1), one of several kinases with TDP-43 affinity, suppresses TDP-43-related toxicity *in vivo* (Salado et al., 2014); while phosphorylation at Ser409/410 shows resistance to cleavage of exogenous TDP-43 increasing self-aggregation and serving as the seed for inclusions (Yamashita et al., 2016).

Similar to phosphorylation, TDP-43 ubiquitination is long considered the main hallmark of pathological protein aggregation since it is often found in FTLD and ALS brain inclusion bodies (Neumann et al., 2006). This ubiquitination affects the overall TDP-43 protein concentration through activation of UPS and autophagy degradation pathways (Scotter et al., 2014), and alters TDP-43 half-life, subcellular localization, and protein aggregation possibly contributing to TDP-43 mediated neurotoxicity (Prasad et al., 2019).

One of the more prominent hallmarks of TDP-43-dependent neuropathology is the presence of TDP-43 C-terminal fragments (CTFs) generated by caspases and calpain proteases (Yang et al., 2010; Zhang et al., 2013). Overexpression of CTFs between 15 kDa and 35 kDa often recapitulate many of the pathological features of TDP-43 proteinopathies and they are commonly detected *in vitro* and *in vivo* (Chhangani et al., 2021). For instance, expression of CTF-25 in neural cells resulted in co-aggregation with full-length TDP-43 and impaired neurite growth in the mouse motor neuron-like hybrid cell line NSC-34 (Yang et al., 2010). Moreover, in a conditional mouse model, accumulation of truncated TDP-43 led to hippocampal neurodegeneration and increased phosphorylation of endogenous full-length TDP-43, while downregulation of CTFs resulted in an alleviation of the pathological phenotype (Walker et al., 2013). Altogether, these data suggest that generation or overexpression of CTFs could exert a toxic role in TDP-43-related proteinopathies. Curiously, proteolytic cleavage of TDP-43 has also been proposed as an adaptive response to exceeding optimum TDP-43 protein levels (Li et al., 2015; Berning and Walker, 2019). For example, TDP-43 mutations resistant to caspase-cleavage (D89E and D169E) triggered cell death more rapidly than wild-type TDP-43 *in vitro*. Meanwhile, the initiation of early-stage caspase-mediated TDP-43 cleavage suppressed TDP-43 related toxicity in cell cultures (Suzuki et al., 2011). In addition, efforts to enhance CTF degradation by upregulating various chaperones, autophagy and UPS, resulted in a reduction of TDP-43 aggregation and formation of inclusion bodies (Cicardi et al., 2018; Kitamura et al., 2018).

### ER Stress and UPR

The endoplasmic reticulum (ER) is a membranous organelle largely responsible for protein maturation within the cell. Proteins that pass through the ER undergo post-translational modifications including glycosylation, oligomerization, disulfide bond formation, pro-isomerization, and subsequent folding with the help of resident ER chaperones, glycosylating enzymes, and oxidoreductases (Braakman and Hebert, 2013). Interestingly, perturbations of protein maturation mechanics are common during normal physiology. Although the calcium-rich environment of the ER lumen is well suited for protein maturation, the success rate of proper folding is estimated to be under 20%. To prevent misfolded proteins from aggregating within the lumen, the ER is readily equipped with ER-associated degradation (ERAD) machinery that recognizes, ubiquitinates, and relocates proteins to the cytoplasm to be degraded by the proteasome (Oakes and Papa, 2015). However, certain conditions including disruption of ER-calcium homeostasis, proteostasis imbalance, and hypoxia among others, may overwhelm protein-folding capacity as described by the term ER stress.

**Figure 2:**
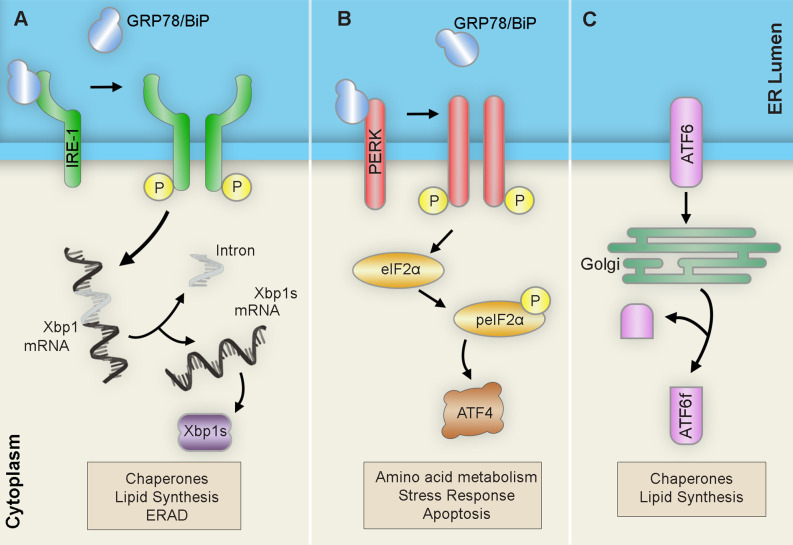
Unfolded protein response (UPR) pathways. Upon ER stress, the ER launches three pathways collectively known as the unfolded protein response (UPR) in an attempt to restore proteostasis: **(A)** IRE1 homodimerizes and autophosphorylates to promote the alternative splicing of Xbp1 mRNA into the Xbp1 spliced form (Xbp1s) transcript. After translation, the resulting Xbp1s transcription factor translocates to the nucleus of the cell to activate ER chaperones, ERAD components, and lipid biosynthesis factors. **(B)** PERK homodimerizes and autophosphorylates to induce eIF-2α phosphorylation ultimately leading to the upregulation of the transcription factor ATF4. This will result in subsequent upregulation of amino acid metabolism, stress response, and apoptosis. **(C)** ATF6 translocates to the Golgi where it is cleaved into its active form ATF6f and then moves to the nucleus to trigger the expression of ER chaperones, XBP-1, CHOP, and members of the ER-associated degradation (ERAD) pathway. Abbreviations: ATF, activating transcription factor; ATF6f, activating transcription factor 6 fragment; CHOP, C/EBP-homologous protein; eIF-2α, eukaryotic translation initiation factor 2α; ERAD, ER-associated degradation; IRE1, inositol requiring transmembrane kinase/endonuclease-1; P, phosphate; PERK, PKR-like ER kinase; XBP-1 unspliced, X-box binding protein 1; XBP1s, XBP-1 spliced.

Upon detection of ER stress, the ER launches three interrelated adaptive pathways collectively known as the unfolded protein response (UPR) to restore proteostasis. There are three UPR pathways named after their respective signalers: Inositol-requiring transmembrane kinase/endoribonuclease 1α (IRE1α), Protein kinase RNA-like endoplasmic reticulum kinase (PERK), and Activating transcription factor 6 (ATF6). These pathways operate in parallel and act to coordinate a series of proadaptive cascades in an effort to restore proper protein folding capacity (Figure 2). However, if stress is irremediable or chronic, the UPR promotes a pro-apoptotic conclusion (Hetz and Papa, 2018).

The first branch of the UPR is mediated by the kinase and endoribonuclease membrane-bound protein sensor, IRE1 (Chen and Brandizzi, 2013). IRE1 regulates survival outcome by signaling the establishment of adaptive or pro-apoptotic cascades. Upon ER stress detection, the IRE1 kinase and endoribonuclease activity is activated by subsequent dimerization and trans-autophosphorylation. The activated RNase domain then readily catalyzes the unconventional splicing of the X-Box binding protein 1 (XBP1) mRNA. The resulting XBP1 spliced (XBP1s) transcript encodes a potent transcription factor that translocates to the nucleus to upregulate adaptive UPR factors like ER chaperones or ER-associated degradation components. However, sustained IRE1 function mediates the activation of signaling cascades that ultimately end in cell death.

Meanwhile, PERK serves to relieve the ER from excessive inputs by attenuating global protein synthesis (Hughes and Mallucci, 2019). Upon ER stress, the ER-resident transmembrane kinase PERK dimerizes and phosphorylates itself, activating its kinase domain and inducing the phosphorylation of target PERK substrates. Among these, the phosphorylation of the eukaryotic initiation factor 2α (eIF2α) effectively halts translation for most messenger RNAs (mRNAs) by preventing the formation of the preinitiation complex at the ribosome. This mechanism allows distinct mRNAs carrying short open reading frames in the 5’-untranslated regions (5’-UTR) to bypass and become enriched. One of these mRNAs encodes the activating transcription factor 4 (ATF4), which operates to upregulate proadaptive genes; however, sustained levels of stress induce the proapoptotic C/EBP homologous protein (CHOP) and growth arrest and DNA damage-inducible protein (GADD34) pathways. Moreover, prolonged attenuation of global protein synthesis carries detrimental implications to cellular proteostasis. Interestingly, stress-mediated phosphorylation of eIF2α triggers the assembly of membrane less cytoplasmic structures known as stress granules (SG), which are often associated with neurodegenerative disorders and are composed of mRNAs, RNA-binding proteins, and ribonucleoproteins (Kedersha et al., 2002).

The last arm of the UPR is mediated by ATF6 (Haze et al., 1999). The ATF6 pathway is primarily implicated in eliciting proadaptive responses important for protein folding, degradation, and ER expansion. ATF6 is generated as an ER-resident transmembrane protein that, upon detection of misfolded proteins, translocates to the Golgi apparatus where it is processed and cleaved generating the active transcription factor, ATF6f. ATF6f then travels to the nucleus and targets ER proadaptive stress response genes such as BiP, protein disulfide isomerase, and glucose-regulated protein 94 (Grp94).

Together, the three branches of the UPR cooperate to restore cellular proteostasis upset by ER stress. The UPR acts to increase the folding capacity of the ER, promotes ER-associated degradation (ERAD), and reduces the burden of protein inputs to mitigate luminal protein accumulation and misfolding. However, when ER stress is unmanageable, the UPR alters its function to promote apoptotic programs. This dynamic function of the UPR in determining cell fate is especially relevant in the context of neurodegenerative diseases characterized by severe proteostatic imbalances.

## TDP-43 and ER Stress

As described above, abnormal accumulation of TDP-43 in the cytoplasm, as well as TDP-43 protein modifications, have largely been associated with the development of several neurodegenerative disorders including ALS and FTLD (Arai et al., 2006; Neumann et al., 2006; Hasegawa et al., 2008; Igaz et al., 2008). Therefore, it is reasonable to suggest that regulatory mechanisms involved in the maintenance of protein homeostasis, such as ERAD and the UPR, could play an important role not only in cell survival but in disease development as well. Initial studies with spinal cord neurons from ALS patients showed that depletion of nuclear TDP-43 correlates with increased mislocalization of TDP-43 to the rough ER suggesting a dynamic relationship between the ER, TDP-43, and neurodegeneration (Sasaki et al., 2010). However, whether TDP-43 mislocalization and aggregation is the culprit, or a byproduct of ER stress is still under debate.

### Does TDP-43 Trigger the UPR?

Activation of ER stress has been documented in tissue samples from ALS and FTLD patients as well as cellular and animal models carrying mutations in familiar ALS-associated genes such as SOD1, VAPB, or FUS (Kikuchi et al., 2006; Gitcho et al., 2009; Saxena et al., 2009; Chen et al., 2010; Langou et al., 2010; Farg et al., 2012). Besides, it is often proposed that TDP-43, WT or mutant, when overexpressed or mislocalized coincides with activation of the ER stress response. However, a closer look into the available literature reveals a more complex story. While some reports insisted on a lack of association, others drew a direct connection between ER stress and TDP-43.

In 2010, Suzuki and colleagues first found that cells that express two-three times the endogenous levels of TDP-43 show increased mRNA levels of C/EBP-homologous protein (CHOP), a protein closely associated with ER-mediated apoptosis whose regulation is mainly mediated through the PERK/eIF2α/ATF4 pathway (Suzuki and Matsuoka, 2012). However, a more careful review revealed that levels of phosphorylated PERK (pPERK), phosphorylated eIF2α (p-eIF2α), ATF4, XBP1s, and cleaved ATF6 remained intact, suggesting that TDP-43-mediated upregulation of CHOP occurred independently of any of the three UPR branches. Instead, the authors suggested that an unidentified role of TDP-43 was responsible for the abnormal regulation of mRNA levels and attenuated degradation of CHOP, independently of UPR sensors involvement (Suzuki and Matsuoka, 2012).

Consistently, the overexpression of TDP-43-WT, mutant M337V, or A382T in HEK293 cells and primary motor neurons also failed to activate the UPR (Mutihac et al., 2015). Here, mutant TDP-43-expressing cells showed an increase in TDP-43 mislocalization to the cytoplasm as well as an increase in protein inclusions compared to cells expressing TDP-43 wild type. However, the levels of the ER-stress resident chaperone GRP-78/BiP, autophagy substrate p62, and ER-stress factor XBP1s were unaltered, indicating a possible lack of correlation between mutant TDP-43 overexpression and induction of ER stress response. Instead, all evidence pointed to the dysregulation of B-cell lymphoma gene 2 (Bcl-2)-mediated ER calcium (Ca^2+^) signaling being responsible for the increase in TDP-43-associated apoptosis since immortalized cells and primary neurons carrying TDP-43-M337V and TDP-43-A382T mutations presented a 50% reduction of luminal ER Ca^2 +^ levels, and delayed Ca^2 +^ flux when compared to TDP-43-WT (Mutihac et al., 2015). In normal conditions, the ER stores high amounts of Ca^2+^, and Bcl-2 has been proposed as a regulator of Ca^2+^ flux (Rong et al., 2008). Thus, the elevated levels of Bcl-2 observed in TDP-43 expressing cells could be associated with changes in the regulation of ER Ca^2 +^ signaling, and partially responsible for the TDP-43-mediated neurotoxicity, acting independently of the UPR (Mutihac et al., 2015).

Forebrain neurons of rats conditionally expressing TDP-43-M337V equally failed to produce evidence of UPR activation despite finding significant signs of Golgi fragmentation, a common hallmark of neurodegeneration, and ubiquitin aggregation, a sign of an overloaded ubiquitin-proteasome degradation system (Tong et al., 2012). Instead, the authors noticed that the levels of XBP1s and XBP1u, although highly upregulated in reactive microglia, appeared significantly reduced in neurons when compared to controls. A hypothesis suggests that TDP-43 overexpression could cause XBP1 deficiency in neurons through an undefined mechanism (Tong et al., 2012). If so, the cell could not activate an IRE-1-mediated ER stress response to deal with TDP-43 toxicity, thereby contributing to the development of pathogenesis (Tong et al., 2012). However, since then, these results have not been replicated nor further evidence has been found supporting that theory.

In contrast, an independent analysis of Neuro2a cells transfected with both WT and mutant TDP-43 (Q331K or A315T) exhibited significantly increased levels of the ER markers XBP1 and ATF6 when compared to control cells (Walker et al., 2013). Despite differences in intensity, these data indicate that both WT and mutant TDP-43 can indeed activate the main UPR pathways. Then, why have previous studies failed to detect the activation of UPR? This may be due, at least in part, to the different TDP-43 mutant species and/or experimental models used in each study. In this regard, Walker and colleagues proposed that the use of single-cell analysis, a more specialized methodology than the ones previously used, allows for a finer quantification of ER markers. In addition, analysis of ER activation at different time points post-transfection may also play a relevant role. For instance, analysis of samples between 18 and 24 h post-transfection showed an increase of ATF6, XBP-1, and CHOP, however, induction of ER stress was highly variable or absent at later time points (Walker et al., 2013).

Walker and collaborators also detected highly upregulated levels of CHOP in cells expressing the two ALS-linked TDP-43 mutants, A315T or Q331K (Walker et al., 2013). Moreover, overexpression of TDP-35 and -25 CTFs (both consistently detected in the frontal and temporal lobes of people with ALS and FTLD; Berning and Walker, 2019) produced an increase in levels of p-eIF2α and CHOP expression as well as cleaved caspase-12 (Wang et al., 2015a), a major ER stress-induced pro-apoptotic protein. These results were further confirmed by Wang and colleagues who found upregulation of the glucose-regulated protein 78 (GRP-78), p-eIF2α, CHOP, cleaved caspase-12, cleaved caspase-3, and cleaved caspase-9, as well as down-regulation of pro-apoptotic Bcl-2 family proteins after overexpressing TDP-43-WT and mutant A315T in neural SH-SY5Y cells (Wang et al., 2015b). Similarly, another study that overexpressed TDP-43 Q331K in SH-SY5Ycells showed higher levels of GRP-78, ATF4, CHOP, protein disulfide isomerase (PDI), and cleaved caspase 12, compared to TDP-43 WT. In addition, TDP-43-Q331K triggered the expression of Beclin1 and p62 and lowered the LC3-II/ LC3-I ratio, a marker of impaired autophagic flux often associated with neurodegenerative disorders (Hu et al., 2019).

### How Does ER Stress Affect TDP-43 Function?

The second pressing question when interrogating the relationship between ER stress and TDP-43 is whether activation of ER stress can protect the cell against TDP-43 toxicity possibly by potentiating stress granules (SG) formation, refolding and degrading misfolded proteins; or, if instead, ER stress promotes TDP-43 mislocalization, post-translational modifications, and aggregation that may accelerate cell death. To address this, several groups investigated the interplay between TDP-43 and ER stress through overexpression paradigms, although experimental procedures relying on pharmacological regulation of ER stress have been the preferred approach.

In 2013, Walker and collaborators used the ER stressor thapsigargin on Neuro2a cells expressing seven different forms of TDP-43 associated with ALS and FTLD (WT, A315T, M337V, D169G, G294A, Q331K, and N390D). The authors observed that while in normal conditions all studied isoforms localized mostly in the nucleus with only moderate cytoplasmic localization after thapsigargin treatment TDP-43 mislocalization to the cytoplasm significantly increased for all TDP-43 variants when compared to untreated cell cultures (Walker et al., 2013). Moreover, when the thapsigargin treatment was combined with salubrinal, to potentiate induction of the PERK pathway by inhibiting eIF2α dephosphorylation, the formation of TDP-43-positive SG was also significantly increased (Walker et al., 2013). These results were confirmed in an independent study that stably expressed TDP-43-WT and TDP-43-A382T also in HEK293 cells, where TDP-43 mislocalization and aggregation significantly increased after exposure to thapsigargin. Interestingly, in this study, the TDP-43 cytoplasmic levels and aggregation in cells expressing TDP-43-M337V were already significantly elevated before drug administration and its subsequent exposure did not cause any further deterioration (Mutihac et al., 2015).

Similarly, treatment with tunicamycin, another potent ER stressor, caused the formation of perinuclear TDP-43 inclusions in motor neuronal cell line NSC-34. Surprisingly, the formation of inclusions was not accompanied by a depletion of TDP-43 from the nucleus. Instead, TDP-43 species appeared to redistribute within the insoluble fraction likely due to the induction of CK1-dependent phosphorylation of TDP-43 (Hicks et al., 2020). This increase in TDP-43 phosphorylation and aggregation did not lead to cell death or metabolic changes suggesting that, at this stage, TDP-43 phosphorylation and cytoplasmic aggregation acted as a protective mechanism against stress instead of being detrimental to the cell. Coincidentally, a previous study performed in human neurons generated from patient fibroblasts carrying the TDP-43-M337V mutant by using induced pluripotent stem cells (iPSCs) failed to detect an increase in neuronal death after treatment with tunicamycin when compared to control cells (Bilican et al., 2012).

Taking all this data together, it would seem that TDP-43, or at least some of its variants, induce the formation of SGs early in the disease process probably as a cell defensive mechanism. For instance, manipulation of genes directly associated with SG formation heavily impacted TDP-43 toxicity in a yeast model causing both enhanced and rescued phenotypes, further connecting SG formation with disease pathogenesis (Kim et al., 2014).

However, when the stress is prolonged in time, chronic SG formation ceases to be protective and turns detrimental to the cell (Bentmann et al., 2013; Li et al., 2013). With this in mind, Kim and collaborators genetically modulated the expression of several genes involved in the regulation of eIF2α phosphorylation to investigate the effect of SG formation over TDP-43 neurotoxicity in the fruit fly. Interestingly, knock-down of the *Drosophila* ortholog for PERK (PEK) caused a decrease in the levels of p-eIF2α leading to a significant improvement of the locomotor deficit caused by TDP-43 expression. Conversely, knocking-down of Gadd34 resulted in increased eIF2α-phosphorylation that translated into exacerbated motility impairment in the fly (Kim et al., 2014). Thus, a prolonged eIF2α phosphorylation and sustained SG formation seemed to play an important role in TDP-43-mediated neurotoxicity. In fact, treatment with GSK2606414, a potent PERK inhibitor, markedly reduced the level of eIF2α-phosphorylation causing a dramatic reduction of TDP-43-induced climbing dysfunction in the *Drosophila* model. This suggests that pharmacological approaches against eIF2α phosphorylation could emerge as a new therapeutic approach for TDP-43 proteinopathies (Kim et al., 2014).

**Figure 3:**
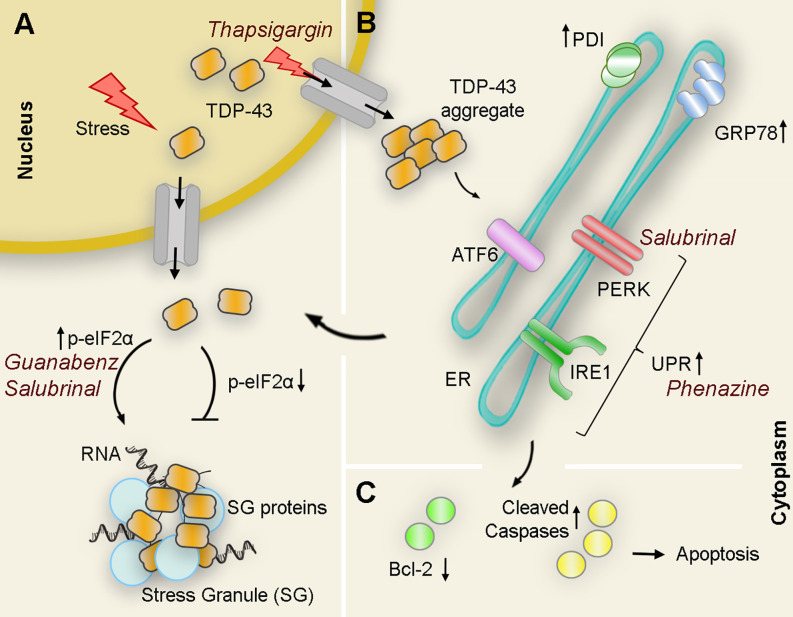
Interplay of TDP43 and ER stress interactions with pharmacological modulators: Under pathological or stressful environmental conditions (i.e., thapsigargin) TDP-43 translocates to the cytoplasm where it forms aggregates or associates with other proteins to form stress granules (SG). **(A)** Upregulation of the PERK substrate p-eIF2α directly correlates with SG formation as pharmacological treatments that maintain levels of p-eIF2α (guanabenz and salubrinal) potentiate ER stress-mediated SG formation. Conversely, genetic down-regulation of peIF2α suppresses the formation of WT and mutant TDP-43-mediated inclusions. **(B)** Overexpression of WT or mutant TDP-43 also results in an increase of cleaved ATF6 and IRE1α substrate Xbp1 as well as ER chaperones PDI and GRP-78 suggesting activation of UPR in the presence of aberrant TDP-43. Pharmacological activation of UPR (i.e., phenazine) decreases TDP-43 insoluble levels. However, chronic activation of ER stress leads to an increase of TDP-43 accumulation in the cytoplasm. **(C)** If TDP-43-related ER stress is prolonged, proapoptotic cascades are induced, antiapoptotic proteins (Bcl-2 family proteins, green circles) are reduced, while antiapoptotic proteins (cleaved caspases, yellow circles) are induced leading to apoptosis. PDI, protein disulfide isomerase.

In a different approach aimed at understanding the specific role of UPR against mutant TDP-43 neurotoxicity, Vaccaro and collaborators focused on three different compounds known to modulate ER stress in transgenic worms and zebrafish models. In this case, administration of Salubrinal (an eIF2α phosphatase inhibitor), guanabenz (an α2-adrenergic receptor agonist that interferes with stress-induced dephosphorylation of eIF2α), and phenazine (a bacterial redox-active exotoxin associated with upregulation of ER stress), resulted in improved motor symptoms and reduced neurodegeneration in both animal models. In correlation with this phenotypical improvement, the authors also detected a significant decrease in the amount of insoluble TDP-43 upon treatment, likely due to an enhancement of protein clearance (Vaccaro et al., 2013). A closer look at the mechanisms involved revealed that each compound preferred one or more specific and distinct branches of the UPR to achieve neuroprotection. While phenazine acted through IRE1 and PERK but independently of ATF6 to reduce TDP-43 proteotoxicity, salubrinal, and guanabenz, both inhibitors of eIF2α phosphatases, favored the PERK branch (Figure 3). Moreover, while the guanabenz positive effect depended partially on IRE-1 and ATF6, suggesting the existence of an eIF2α independent off-target, the protective role of salubrinal occurred completely independent of the IRE1 or ATF6 branches of the UPR (Vaccaro et al., 2013). This opens up the possibility of using a combination of compounds acting at different or overlapping branches of the UPR to enhance therapeutic efficacy.

## Concluding Remarks

The unfolded protein response (UPR) pathways mediated by PERK, IRE1α and ATF6 play a fundamental role in the maintenance of protein homeostasis. This role is even more crucial in the context of neurodegenerative disorders where protein synthesis, refolding and degradation might dictate the fatal outcome of neuronal cells and, therefore, the organism. The accumulation of pathological inclusions composed of TDP-43 aggregates is a common feature of many proteinopathies including amyotrophic lateral sclerosis (ALS) and frontotemporal lobar degeneration (FTLD). Therefore, understanding the specific contribution of TDP-43 to ER stress and how ER stress harnessing could modulate or even alleviate TDP-43 aggregation and toxicity, are two of the most important puzzles in the field. In this regard, we have identified key questions that deserve serious consideration. For instance:

- What is the mechanism by which TDP-43 upregulates CHOP independently of UPR signaling?
- Is there a molecular crosstalk between Ca^2+^ dyshomeostasis, TDP-43, and ER stress (Dafinca et al., 2021)?
- How TDP-43 overexpression triggers neuronal XBP1s depletion in transgenic rats? Are components of the IRE1 branch disrupted or sequestered, in this case, resulting in a lack of protection?
- Is XBP1s absent in the nuclei of neurons from patients displaying TDP-43 pathology?
- Could we enhance protection against TDP-43 toxicity by combining pharmacological modulators of ER stress acting at different branches of the UPR?
- Could we block, delay, or prevent TDP-43 toxicity through orthogonal, stress-independent, activation of one or more UPR branches (Shoulders et al., 2013)?

Several groups have used different approaches and models (both *in vivo* and *in vitro*) to understand the complex relationship between TDP-43 and ER stress over the last decade. Despite initial reports, where the specificity and sensitivity of the assays might have masked or altered the results, it seems that overexpression of mutant forms of TDP-43 and, to a lesser extent, TDP-43-WT might activate the UPR as a protective response mechanism by the cell. In turn, potentiation or pharmacological activation of ER stress could serve as a mechanism to modulate TDP-43-mediated toxicity. However, the inconsistent results between studies might cloud this approach or call for caution. The differences between cell and animal models, TDP-43 transgenes, ER stressors, and even experimental methodology could easily account for many of the observed discrepancies. Moreover, members of the UPR branches are differentially expressed in different cell types (Walter and Ron, 2011), which may also influence the efficacy of the stress response and interpretation of data. Therefore, a more consistent approach considering the different behaviors between TDP-43 species, the use of more physiologically relevant systems such as patient-derived iPSCs, and a better understanding of how models and methodology affect TDP-43 phenotypes and function might be the key to resolve, if not all, at least some of the difficult questions that lie within this complex relationship.

## Author Contributions

LM, JL-S, and DER-L conceived the original idea and revised the manuscript. LM and JL-S designed the outlines of the study, performed literature review, wrote the first draft, and prepared the figures. All authors contributed to the article and approved the submitted version.

## Conflict of Interest

The authors declare that the research was conducted in the absence of any commercial or financial relationships that could be construed as a potential conflict of interest.

## Publisher’s Note

All claims expressed in this article are solely those of the authors and do not necessarily represent those of their affiliated organizations, or those of the publisher, the editors and the reviewers. Any product that may be evaluated in this article, or claim that may be made by its manufacturer, is not guaranteed or endorsed by the publisher.
